# Thermographic Scan of the Thoracolumbar Area in Dogs with Acute Intervertebral Disc Extrusion (IVDE): A Retrospective Study

**DOI:** 10.3390/life15010068

**Published:** 2025-01-09

**Authors:** Cristian Zaha, Liliana Cărpinișan, Larisa Schuszler, Nistor Paula, Tudor Căsălean, Tiana Florea, Văduva Cristina, Bogdan Sicoe, Ciprian Rujescu, Roxana Dascălu

**Affiliations:** 1 Surgery Clinic, Faculty of Veterinary Medicine, University of Life Sciences “King Michael I”, 300645 Timisoara, Romania; cristian.zaha@usvt.ro (C.Z.); larisaschuszler@usvt.ro (L.S.); paula.nistor@usvt.ro (N.P.); Tudor-Mihai.Casalean.FMV@usvt.ro (T.C.); roxanadascalu@usvt.ro (R.D.); 2 Dermatology Department, Faculty of Veterinary Medicine, University of Life Sciences “King Mihai I”, 300645 Timisoara, Romania; tijana.florea@usvt.ro; 3 Internal Medicine, Faculty of Veterinary Medicine, University of Life Sciences “King Michael I”, 300645 Timisoara, Romania; cristina.vaduva@usvt.ro; 4 Diagnostic Imaging, Faculty of Veterinary Medicine, University of Life Sciences “King Michael I”, 300645 Timisoara, Romania; bogdan.sicoe@usvt.ro; 5 Management and Rural Development Department, Faculty of Management and Rural Tourism, University of Life Sciences “King Michael I”, 300645 Timisoara, Romania; rujescu@usvt.ro

**Keywords:** dogs, intervertebral disc extrusion, thermography

## Abstract

Background: several authors have documented variations in local temperature in both horses and dogs presenting acute intervertebral disc extrusion (IVDE) along the entire spinal column. However, none have demonstrated distinct temperature differences between healthy animals and those with IVDE. A retrospective study was conducted to assess the efficacy of thermography at evaluating local temperature and thermal patterns in healthy dogs as well in those with IVDE across the T11–L3 area. Methods: the study included 20 healthy dogs and 32 dogs with IVDE. For both groups of dogs, the thoracolumbar region was trimmed and, subsequently, scanned using the Flir E50 thermography device. The Flir Tool software was used to analyze three designated areas (Bx1, Bx2, Bx3) within the thoracolumbar region by comparing the average temperature of the minimum, maximum, and mean temperature recordings between the two groups. Results: the thermal pattern and the local temperature of the thoracolumbar area present differences between healthy dogs and those with IVDE. Conclusions: we recommend thermographic scanning of the thoracolumbar area to find differences in local temperature between healthy dogs and those with intervertebral disc extrusion. Further investigations are required to differentiate between disc extrusion that exhibits lateralization to the right or left.

## 1. Introduction

Intervertebral disc degeneration is a common disease and one of the most serious problems worldwide in chondrodystrophic breeds [1]. Intervertebral disc disease (IVDD) in the thoracolumbar region is the leading cause of paraplegia in chondrodystrophic dogs, commonly occurring between 3 and 7 years of age [1,2,3]. Several authors have identified a higher frequency of disc extrusion in the T11–L3 region in dogs, as this is the most mobile area during running. This increased mobility contributes to heightened mechanical stress on the intervertebral discs, ultimately culminating in their progressive degeneration [4]. The likelihood of successful recovery is closely tied to the speed of diagnosis and treatment following disc material extrusion. Intervertebral disc injuries are caused by aging and mechanical stress, both of which contribute to degenerative changes in the spine [1,5]. Intervertebral disc disease is divided into acute intervertebral disc extrusion (IVDE) and intervertebral disc protrusion [3,6]. Also, a genetic factor is involved in IVDE, as independent genome-wide association studies for skeletal dysplasia and IVDE have identified a highly expressed *FGF4* retrogene on CFA12 which is associated with both IVDE and chondrodystrophy in dogs [7].

The clinical signs of IVDD in dogs depend on the type and location of the disc extrusion and may range from pain and discomfort to severe neurological deficits [8,9]. Types of clinical signs vary from spinal hyperesthesia, ataxia, paraparesis, paraplegia with or without deep pain perception, and urinary and fecal incontinence [1,9].

Various costly methods are available to diagnose intervertebral disc herniation, and most require general anesthesia to obtain clear imaging. Radiography (Rx), myelography, computed tomography (CT), and magnetic resonance imaging (MRI) are used for diagnosis of the intervertebral disk [10,11,12]. This obtained images can be conventional or may require the administration of the contrast solution in the subarachnoidal space in case of IVDE [13,14]. CT provides more precise information and is faster than myelography, especially in chondrodystrophic breeds. A study involving 20 dogs compared the accuracy of CT and myelography in diagnosing acute intervertebral disc disease [13]. The findings revealed that CT was 90% accurate, while myelography was 88% accurate in identifying the primary location of disc herniation [13,15]. CT also correctly predicted the lateralization of disc material in 96% of the cases, compared to 92% for myelography [11]. MRI, with an accuracy of 98.5% in detecting IVDE [16] and also providing transverse imaging, outperforms CT when the disc material is not mineralized, and, in such cases, a contrast medium may be needed [11,17]. However, using CT with a subarachnoid contrast medium negates the advantage of diagnosing disc herniation without the side effects associated with myelography [18].

Medical infrared imaging, also known as infrared thermography (IRT), is a non-invasive method that captures infrared radiation emitted from the body’s surface, creating a visual representation of body temperature. It can also identify abnormal physiological changes in both humans and animals [19,20,21]. The diagnostic accuracy of infrared thermography (IRT) for detecting spinal disease in humans is 80%, while it demonstrates a 90% accuracy in distinguishing normal dogs from those affected by IVDE [21,22,23].

IRT can be a useful tool for the evaluation of body surface temperature changes in response to exercise [19,20], for diagnosing diseases affecting the canine locomotor system [24,25], and for assessing the response of an organism’s body to drug administration [23]. The highest surface temperatures of the body have been observed in the trunk and lumbosacral area, with a temperature gradient across the limbs, where proximal regions are warmer than the distal areas [26].

In human medicine, the normal thermogram of a human spine has been characterized by a central zone of decreased heat emission in the region of the spinal processes from the cervical to the lower lumbosacral spine [27,28]. Pathological alterations in the thermal pattern include focal changes in temperature over the site of an active lesion as well asymmetrical hyperthermia and hypothermia distal to the lesions [29].

Turner T. reported the identification of a localized region of hyperthermia adjacent to the affected disc in horses exhibiting neurological injuries [30].

Grossbard et al. aimed to assess the efficacy of infrared thermography (IRT) in identifying dogs with intervertebral disc disease (IVDD) and evaluate whether the normal thermal pattern is restored 10 weeks following decompression surgery [21].

Given that an increase in local tissue temperature typically follows an inflammatory process, the objective of this study is to compare local temperature measurements and evaluate thermal patterns between healthy dogs and those affected by IVDE.

We hypothesize that the local temperature and thermal pattern of the thoracolumbar area will exhibit significant changes, with an increase in local temperature and a modified thermal pattern in dogs with IVDE.

## 2. Materials and Methods

A retrospective study was performed at the Surgery Clinic of the Faculty of Veterinary Medicine of Timisoara between March 2021 and October 2024.

### 2.1. Animal Selection and Clinical Examination

During this period, 75 dogs from chondrodystrophic breeds (French bulldog, dachshund, bichon frise, Lhasa apso, shih tzu, chihuahua, cocker spaniel, Havanese, and mixed breeds), aged between 3 years and 2 months and 7 years and 4 months and weighing between 9 kg and 15 kg, presented with an acute form of paraplegia. The inclusion criteria for the study were as follows: localization of disc extrusion in the T11–L3 region, presentation for clinical examination within 12 h of paraplegia onset, paresis with decreased and absent proprioception, paralysis with deep pain perception present, absence of urinary or fecal incontinence, and rectal temperature between 38 and 39.5 °C. The exclusion criteria were as follows: weight exceeding 15 kg, presence of disc extrusion outside the T11–L3 area, presence of two or more disc extrusions in the examined region, spondylosis in the T11–L3 or L7–S1 segments, discospondylitis administration of anti-inflammatory medication at another clinic or private practice, urinary or fecal incontinence, presence of hemivertebrae, tumors, or thromboembolism.

A clinical and neurological examination was performed by two veterinarians (Z.C. and D.R.), both with clinical experience in musculoskeletal disorders, who were not blinded to each other’s results. The neurological examination included testing proprioceptive deficits in the hind limbs, cutaneous trunchi reflex, patellar reflex, anal reflex, and superficial and deep pain sensation [3]. Following the neurological examination, each dog was assigned a neurological score based on a modified version of the scale published by Wheeler and Sharp [4].

A control group, consisting of 30 dogs from chondrodystrophic breeds (French bulldog, dachshund, shih tzu), underwent clinical and neurological examinations performed by the same veterinarians (Z.C. and D.R.). The inclusion criteria for the control group required the absence of anti-inflammatory drug administration, no reported history of lameness within the preceding month, no radiographic changes to the spinal vertebrae, and a rectal temperature between 38 and 39.5 °C.

### 2.2. Thermal Imaging and Data Recording

Following the clinical examination, the hair in the dorsal region of the animal, covering the T7–L7 segment, was removed using a groomer clipper, both for the dogs in the study group and for those in the control group.

**Figure 1:**
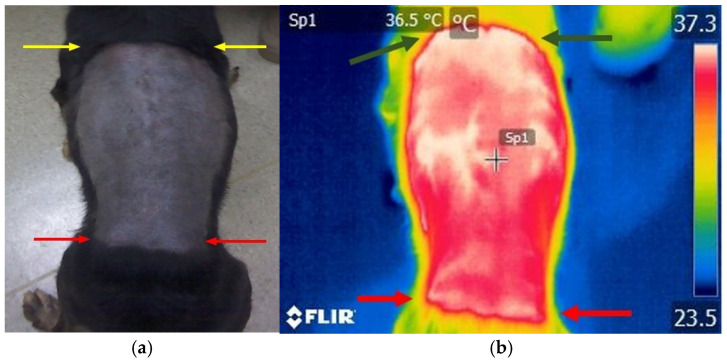
Photo of the thoracolumbar area in a paraplegic dog: (**a**) normal image from the FLIR E50 camera, (**b**) thermographic image from the FLIR E50 camera. Arrows: (**a**) yellow arrows—T7 level, red arrows—L7 level; (**b**) green arrows—T7 level, red arrows—L7 level.

Following hair removal, the animals were allowed a 30 min acclimatization period under controlled environmental conditions, specifically an air temperature of 20–21 °C, humidity between 70% and 75%, and no air currents within the room. Thermographic imaging was performed by the same individual on both the dogs with disc extrusions and those in the control group. The operator was positioned 1 m from the dog at a 90° angle to the thoracolumbar region. Thermographic images (Figure 1a,b) were obtained using the FLIR E50 thermography device (FLIR Systems Inc., Wilsonville, OR, USA), with the following parameters: 0.95 emissivity and a resolution of 240 × 180 for each thermographic image. The temperature range was set from −20 °C to 650 °C, with a sensitivity of ≤0.05 °C.

The obtained thermographic images were processed and interpreted by the same operator, using the FLIR Tools software 5.X for image analysis. For the interpretation of the thermographic images, a rectangle (Bx1, Bx2, and Bx3) with dimensions of 94 × 42 pixels was drawn.

For the dogs in the control group, thermographic images were obtained between 9 a.m. and 2 p.m., while, for the dogs in the study group, thermographic images were obtained between 9 a.m. and 4 p.m., depending on their presentation for the clinical examination.

### 2.3. Imagistic Investigations

The diagnosis of the spinal cord compression was established after a radiological exam and a CT scan. The radiological exam required two views (latero-lateral and ventro-dorsal) using the Siemens Multix Swing device. For the CT scan, the animal required anesthesia by administering to the animal a combination of medetomidine in doses of 0.02 mg/kg (Domitor 2%, Montero, Romania) and ketamine in doses of 5 mg/kg (Ketamidor 100 mg/mL, Richter Pharma, Austria), and, if necessary, it was supplemented with propofol in doses of 4 mg/kg (Propofol 10 mg/mL, Braun, Romania).

### 2.4. Statistical Analysis

Two groups of dogs were subjected to a comparative analysis of the values obtained through thermographic scanning, which included a control group (*n* = 20) and a study group (*n* = 32). For each case, three rectangular regions centered on the thoracolumbar area, labeled Bx1, Bx2, and Bx3, were examined. This resulted in a set of statistical data derived from the observed temperatures, which were expressed in degrees Celsius. For each thermographic image, the mean of the maximum, minimum, and average temperature values recorded within the designated rectangle in the FLIR Tools software was considered. The comparisons involved examining the differences between the study group and the control group, based on the location from which the data were obtained: Bx1 first, then Bx2, and, separately, Bx3. Additionally, the means of the minimum, maximum, and average values were compared separately. This resulted in nine comparisons for 18 datasets. A two-sample *t*-test was performed using SAS Studio, following normality testing of the distributions with the Shapiro–Wilk test.

## 3. Results

### 3.1. Animal Information

Out of 75 consecutively examined cases, including clinical and neurological exams, 47 cases were selected for radiographic imaging (27 males and 20 females). After the exclusion criteria were applied to the examined dogs, twenty-eight of them were excluded for the following reasons: four dogs weighed over 15 kg, six dogs had chronic paralysis, 10 dogs presented without deep pain perception, and eight dogs presented with urinary or fecal incontinence.

Following the clinical and neurological examinations, five dogs were classified with grade 1 deficits, 19 dogs with grade 2 deficits, 14 dogs with grade 3 deficits, eight dogs with grade 4 deficits, and one dog with grade 5 deficits. All results were obtained using the scale of Wheeler and Sharp [4].

Following radiographic imaging, 39 cases were selected for computed tomography (CT). A total of eight dogs were excluded from the study due to the following reasons: spondylosis in the L7–S1 region (two dogs), T13–L1 region (three dogs), L1–L2 region (one dog), and L2–L3 region (two dogs). The dogs that met the inclusion criteria were aged between 3 and 8 years (mean age: 4.8 years), with a mean weight of 8.55 kg (range: 4.32–14.80 kg). The study included the following breeds: six French bulldogs, 10 dachshunds, seven bichon frises, two Lhasa apsos, six shih tzus, two chihuahuas, two cocker spaniels, one Havanese, and three mixed breeds.

Out of the 30 dogs in the control group, 20 dogs were included in the study. The excluded dogs did not meet the following criteria: four dogs exhibited pain upon palpation in the dorsal lumbar region, two dogs showed spondylosis in the T13–L1 and L1–L2 regions, and four other dogs presented spondylosis in the L7–S1 region. The dogs in the control group belonged to the following breeds: shih tzu (five dogs), dachshunds (eight dogs), bichon frise (four dogs), cocker spaniels (three dogs). They were aged between 4 and 7 years (mean age: 5.2 years), with a mean weight of 9.35 kg (range: 5.4–14.2 kg).

The rectal temperature of all dogs was between 38 and 39.5 °C and was measured at the start of the examination.

### 3.2. Imagistic Investigation

A total of 52 conventional radiographs were performed for the study group (*n* = 32) and the control group (*n* = 20). Both ventro-dorsal and latero-lateral views were taken. Following the radiographs, lesions that could be associated with disc extrusion were identified, such as the narrowing of the intervertebral space in seven dogs and increased opacity of the intervertebral foramen in three dogs.

**Figure 2:**
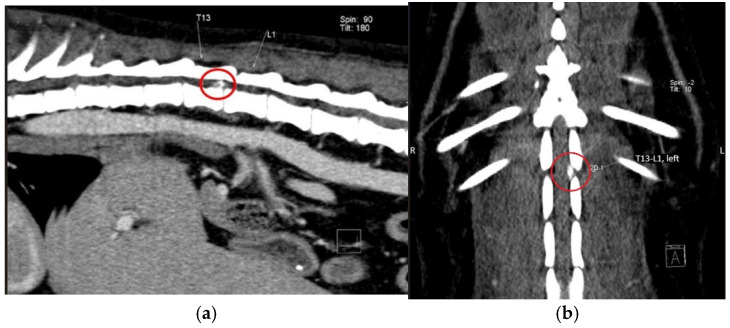
Computed tomography image from a dog with T13–L1 disc extrusion: (**a**) sagitally reconstructed view of the vertebral column, T13—13th thoracic vertebra, L1—1th lumbar vertebra, (**b**) dorsally reconstructed view of the vertebral column, T13—13th thoracic vertebra, L1—1th lumbar vertebra, red circle—extruded discal material.

A total of 39 computed tomography scans were selected, and the results identified the following locations: four in the T11–T12 space, six in the T12–T13 space, 12 in the T13–L1 space (Figure 2a,b), six in the L1–L2 space, and four in the L2–L3 space. In nine cases, the disc material was located beneath the spinal cord, in 12 cases the disc material was identified with left lateralization, and, in 11 cases, the disc material was located on the right side. For seven conventional computed tomography scans, no disc extrusions were identified, and these cases were, therefore, excluded.

A total of 32 cases were selected following neurological, radiographic, and computed tomography examinations for the thermographic imaging study.

### 3.3. Thermography Scan

#### 3.3.1. Thermography Scan of the Control Group

**Figure 3:**
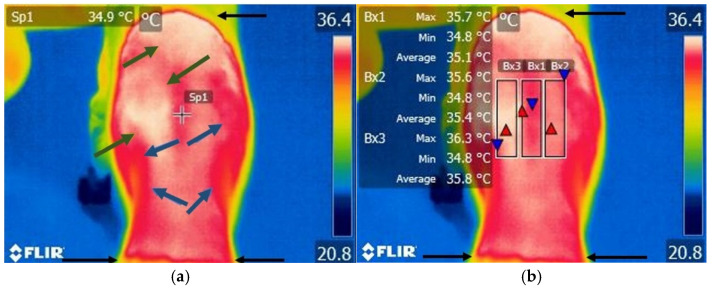
Thermographic image of the thoraco-lumbar area of a healthy dog. (**a**) Scanning image without FLIR Tools software analysis: black arrow—clipped hair area between the T7 and L7 level, green arrow—variation areas of increase in temperature, blue arrow—variation areas of low temperature, spot—local temperature of the skin. (**b**) Scanning image with FLIR Tools software analysis: black arrow—clipped hair area between the T7 and L7 level, Bx1—area of interest centered on the vertebrae, Bx2—area of interest on the right side of the vertebrae, Bx3—aria of interest on the left side of the vertebrae; red triangle spot—maximum temperature recorded in the interest area; blue triangle spot—minimum temperature recorded in the interest area.

Different color variations were observed in the clipped area between T7–L7. There were observed alternations in local temperature, with warmer or cooler areas in all dogs of the control group (Figure 3a,b).

#### 3.3.2. Thermography Scan of the Study Group

**Figure 4:**
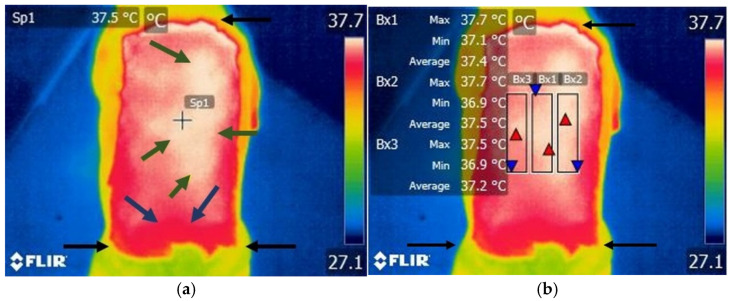
Thermographic image of the thoraco-lumbar area in a dog with T13–L1 disc extrusion. (**a**) Scanning image without FLIR Tools software analysis: black arrow—clipped hair area between the T7 and L7 level, green arrow—area of increased temperature, blue arrow—area of lower temperature, spot—local temperature of the skin. (**b**) Scanning image with FLIR Tools software analysis: black arrow—clipped hair area between the T7 and L7 level, Bx1—area of interest centered on the vertebrae, Bx2—area of interest on the right side of the vertebrae, Bx3—area of interest on the left side of the vertebrae; red triangle spot—maximum temperature recorded in the interest area; blue triangle spot—minimum temperature recorded in the interest area.

A high intensity temperature area was observed in the T11–L3 area in all dogs from the study group. Areas with cooler temperatures were observed at the margin of the T11–L3 area (Figure 4a,b).

### 3.4. Group Comparison

#### 3.4.1. Comparisons Within the Bx1 Region

**Figure 5:**
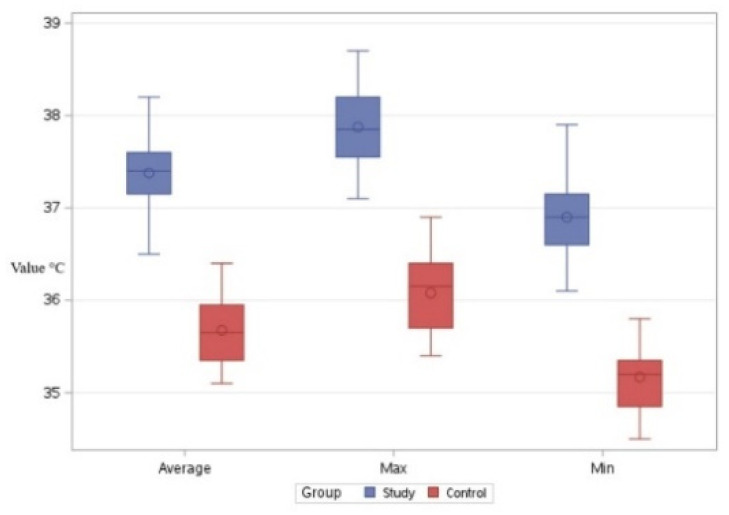
Comparative boxplot referring to the results obtained for Bx1 between the study group (blue mark) and the control group (red mark); distribution of the average, maximum, and minimum temperatures; ◦—outliers.

Regarding the comparisons in Bx1, the series consisting of the minimum values for the control group resulted in an average reading of 35.17 °C. The series consisting of the minimum values for the study group yielded an average result of 36.9 °C. The differences were statistically significant, with a t-value of −15.29 and *p* < 0.001. This indicates that, with respect to these values, animals in the study group exhibited higher local thermal values compared to those in the control group (Figure 5).

Comparing the two groups regarding the maximum temperatures observed in the Bx1 region, an average temperature of 36.08 °C was determined for the control group and an average temperature of 37.87 °C was determined for the study group. In this case as well, the local temperatures in the study group were higher than those in the control group. The differences were statistically significant, with a t-value of −15.46 and *p* < 0.001 (Figure 5).

Comparing the two groups with respect to the average temperature recorded in the Bx1 region, an average of 35.64 °C was observed in the control group and an average of 37.38 °C was observed in the study group. In this case as well, the local temperatures in the study group were higher than those in the control group, with statistically significant differences, t = −18.90, *p* < 0.001 (Figure 5).

#### 3.4.2. Comparisons Within the Bx2 Region

**Figure 6:**
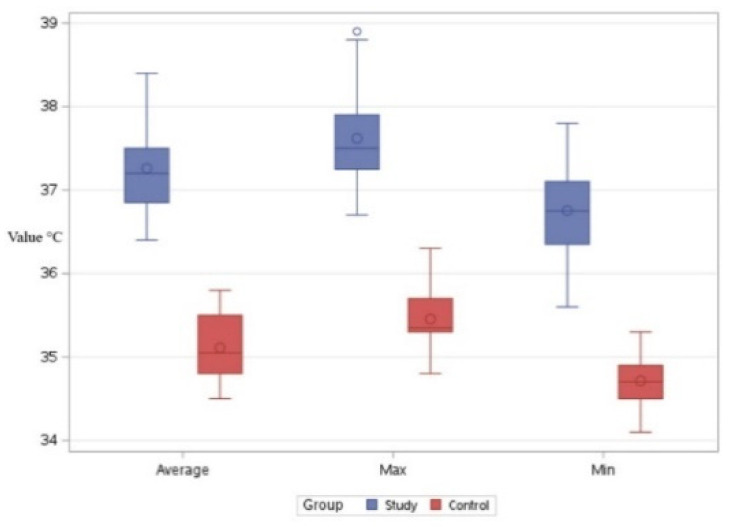
Comparative boxplot referring to the results obtained for Bx2 between the study group (blue mark) and the control group (red mark); distribution of the average, maximum, and minimum temperatures; ◦—outliers.

Regarding the comparisons in Bx2, the series consisting of the minimum values for the control group resulted in an average reading of 34.71 °C. The series consisting of the minimum values for the study group yielded an average result of 36.75 °C. The differences were statistically significant, with a t-value of −15.70 and *p* < 0.001. This indicates that, with respect to these values, animals in the study group exhibited higher local thermal values compared to those in the control group (Figure 6).

Comparing the two groups with respect to the maximum temperatures observed in the Bx2 region, an average of 35.45 °C was determined for the control group and an average of 37.61 °C was determined for the study group. In this case as well, the local temperatures in the study group were higher than those in the control group. The differences were statistically significant, with a t-value of −15.39 and *p* < 0.001 (Figure 6).

Comparing the two groups with respect to the average temperature recorded in the Bx2 region, an average of 35.10 °C was observed in the control group and an average of 37.25 °C was observed in the study group. In this case as well, the local temperatures in the study group were higher than those in the control group, with statistically significant differences, t = −15.76, *p* < 0.001 (Figure 6).

#### 3.4.3. Comparisons Within the Bx3 Region

**Figure 7:**
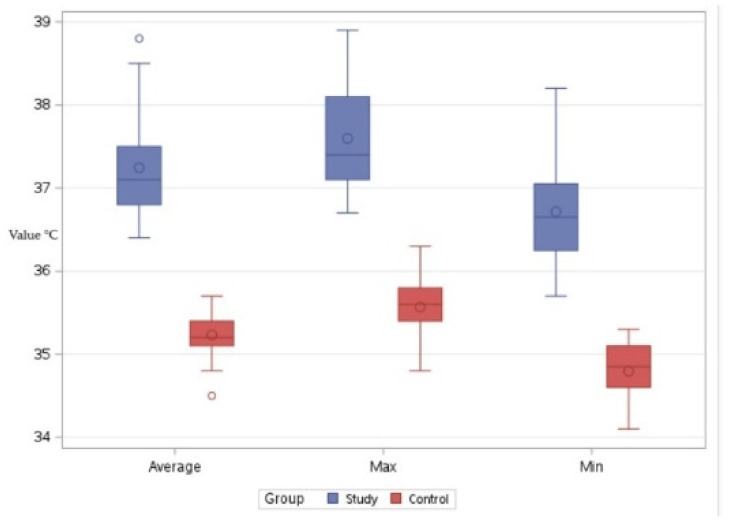
Comparative boxplot referring to the results obtained for Bx3 between the study group (blue mark) and the control group (red mark); distribution of the average, maximum, and minimum temperatures; ◦—outliers.

Regarding the comparisons in Bx3, the series consisting of the minimum values for the control group resulted in an average reading of 34.79 °C, while the minimum values for the study group yielded an average result of 36.71 °C. The differences were statistically significant, with a t-value of −13.51 and *p* < 0.001. This indicates that, with respect to these values, animals in the study group exhibited higher local thermal values compared to those in the control group (Figure 7).

Comparing the two groups with respect to the maximum temperatures observed in the Bx3 region, an average of 35.56 °C was determined for the control group and an average of 37.59 °C was determined for the study group. In this case as well, the local temperatures in the study group were higher than those in the control group. The differences were statistically significant, with a t-value of −13.54 and *p* < 0.001 (Figure 7).

Comparing the two groups with respect to the average temperature recorded in the Bx3 region, an average of 35.23 °C was observed in the control group and an average of 37.24 °C was observed in the study group. In this case as well, the local temperatures in the study group were higher than those in the control group, with statistically significant differences, t = −14.09, *p* < 0.001 (Figure 7).

## 4. Discussion

Thermographic scanning of the thoraco-lumbar region provides important data to differentiate dogs with IVDE from healthy dogs; an aspect that is highlighted is the confirmation of the hypothesis regarding the differences among the minimum, maximum, and mean temperatures considered from the Bx1, Bx2, and Bx3 areas, which were identified in both groups of dogs. The results identified after scanning the healthy dogs and those with IVDE revealed that the values of temperature from the selected areas and the thermal patterns obtained presented changes, with increased temperatures in IVDE dogs.

Dogs with acute intervertebral disc extrusions presented a larger area of increased temperature in the thoraco-lumbar region compared with the same thermal pattern analyzed in healthy dogs. Furthermore, the temperature values recorded in the region of interest (T11–L3) were elevated by more than 1 °C in the study group when compared to the control group. Based on a study performed by Lahiri et al. [31], a local temperature that is 0.7–1.0 higher compared with the surrounding tissue is attributed to an inflammation.

A study conducted by Turner on horses with neurological injuries identified an area of hyperthermia at the site of the injury [30]. Similar findings, including an increase in local skin temperature, have been observed in humans with cervical intervertebral disc disease (IVD), providing valuable data not only for localizing disc herniation but also for detecting its presence [31]. It is crucial to differentiate between temperature increases associated with IVDE and those resulting from other pathological conditions, as thermographic scans alone cannot identify the underlying cause of changes in the thermal pattern, such as tumors or inflammation [3].

Intervertebral disc herniation is classified into Hansen type I and Hansen type II categories [3,32]. Hansen type I herniation predominantly occurs in chondrodystrophic breeds, such as dachshunds, and is typically associated with more severe clinical manifestations compared to Hansen type II [33]. In Hansen type I, there is often acute or subacute extrusions of the nucleus pulposus from a chondroid disc, represented by more severe neurological symptoms [1,7,14]. In our study, the majority of the dogs affected by disc extrusion Hansen type I where from the dachshund breed and presented clinical signs such as acute paralysis and thoraco-lumbar pain. The clinical signs of IVD vary from spinal hyperesthesia to paraplegia with or without proprioception and paralysis with or without deep pain perception [1]. In our study, the most frequent clinical sign was represented by paralysis with deep pain perception. In a study performed by Coates et al., more than 53% of the cases were dachshunds [1].

In our study, we designated the region T11–L3 as the area of interest, as this region is the most affected by disc extrusion. Wheeler and Sharp [4] noticed that, in relation to thoraco-lumbar disc disease, over 50% of the lesions were found at the T12–T13 and T13–L1 discs, while more than 85% occurred between the T11–T12 and L2–L3 levels [32]. The most common site affected by disc extrusion in our study was the T13–L1; a similar observation was made also by other authors in their study [14].

Radiographic evaluation allowed us to identify changes in 10 dogs, e.g., the narrowing of the intervertebral space and the increased opacity in the vertebral foramen; similar observations have been made by others authors in their studies [1,32]. Compared with radiography, CT scans can acquire a diagnostic index without an invasive procedure, require a short imagining time compared with nuclear magnetic resonance imaging, and enable a detailed visualization of the extruded disc material compared with radiography examination [6,10]. In our study, thirty-two disc extrusions were identified through conventional CT, but, in seven cases, conventional CT did not detect the extrusion of the disc material. Some authors have noted that myelography is necessary in situations where the nucleus pulposus does not degenerate or mineralize before extruding and compressing the spinal cord [14,27,33]. Conversely, when the extruded material is not mineralized and no other signs of disc extrusion are present, like the vacuum phenomenon, vertebral endplate sclerosis, and disc space narrowing, a myelography or CT myelography is required for accurate diagnosis and proper localization [6,34].

During the thermographic measurements, the ambient temperature in the examination room was maintained at 21 °C. Each dog, both in the control group and in the study group, underwent an acclimatization period of 30 min. The thoraco-lumbar region was not touched by the operator for at least 30 min prior to the scan. During handling, one hand was placed on the abdominal region and the other on the inguinal region. A temperature of 21 °C has also been cited by other authors as not influencing local skin temperature or thermoregulation in animals [35,36,37]. To prevent artifacts in the thoraco-lumbar area, other authors have also employed similar handling techniques to avoid contact with the area of interest [24,38,39].

Presence of the subcutaneous fat tissue in the L3–L5 vertebral area could have an insulating effect that reduces heat loss through the skin, appearing on thermographic images as an alternation in local temperature [40].

Numerous studies have been conducted to explore the impact of individual animal characteristics on IRT outcomes in healthy animals. Temperature differences between adjacent haired and non-haired skin indicate that the hair absorbs a portion of the radiated heat, thereby preventing this energy from being detected by an infrared camera [41]. Skin color can influence IRT readings, as black areas are typically warmer than adjacent white areas. In zebras, for example, black stripes are warmer than the white stripes during the day, likely due to the increased absorption of solar energy by the black stripes [42].

Circadian, infradian, and ultradian rhythms influence body temperature and should be considered when planning IRT or interpreting thermographic results [43]. In our study, the IRT measurements were performed in the first part of the day.

Thermography has proven to be valuable in human and equine medicine, as well as in diagnosing musculoskeletal injuries in companion animals [44,45,46]. Unlike other imaging techniques, it is non-invasive, it does not require anesthesia, and it does not expose the patient to radiation [24,44]. Numerous studies have highlighted thermography’s ability to detect changes in thermal patterns prior to the appearance of clinical or radiographic signs [47].

The results obtained indicate an elevated temperature in the thoraco-lumbar region in dogs with intervertebral disc extrusion compared to healthy dogs, with these temperature changes associated with local inflammation and muscular spasms. Further investigations are needed to (1) compare the temperature on the left and right sides of the spine to identify correlations with the lateralization of disc extrusion, (2) compare the temperatures observed in disc extrusion pathologies with those associated with other intervertebral disc diseases, (3) assess differences in local temperatures between dogs with acute disc extrusion and those with chronic disc protrusion, and (4) compare differences in local temperature between haired and non-haired dogs with IVDE.

The limitations of the study included the different breeds of dogs taken into consideration, the different distributions between dog breeds, not cutting the hair, the skin color of the dogs, the absence of myelography to identify a disc extrusion without mineralization, the different distribution of the adipose tissue in the thoraco-lumbar area and the moderate resolution of the thermographic device. The operator was not blinded during thermographic scanning or when marking the selected areas for the study.

## 5. Conclusions

The local temperature of the thoraco-lumbar area in dogs with IVDE is higher than the local temperature of the same identified area in healthy dogs.

The thermal pattern of the thoraco-lumbar area present differences in dogs with IVDE compared with healthy ones.

We recommend thermographic scanning of the thoraco-lumbar area as a minimally invasive method to distinguish dogs with IVDE from healthy ones, particularly in hairless dogs.

Further investigations are required to differentiate between disc extrusion that exhibits lateralization to the right or left.

## Data Availability

All the data obtained for this pilot study are available in the Clinical Register of Companian Animals of the Surgery Clinic.
